# Convolutional Neural Networks to Estimate Dry Matter Yield in a Guineagrass Breeding Program Using UAV Remote Sensing

**DOI:** 10.3390/s21123971

**Published:** 2021-06-09

**Authors:** Gabriel Silva de Oliveira, José Marcato Junior, Caio Polidoro, Lucas Prado Osco, Henrique Siqueira, Lucas Rodrigues, Liana Jank, Sanzio Barrios, Cacilda Valle, Rosângela Simeão, Camilo Carromeu, Eloise Silveira, Lúcio André de Castro Jorge, Wesley Gonçalves, Mateus Santos, Edson Matsubara

**Affiliations:** 1 Faculty of Computer Science, Federal University of Mato Grosso do Sul, Campo Grande 79070900, Brazil; silva.eng.gabriel@gmail.com (G.S.d.O.); cpolidoro@gmail.com (C.P.); lucas.rodrigues@ifms.edu.br (L.R.); wesley.goncalves@ufms.br (W.G.); edsontm@facom.ufms.br (E.M.); 2 Faculty of Engineering, Architecture and Urbanism and Geography, Federal University of Mato Grosso do Sul, Campo Grande 79070900, Brazil; lucasosco@unoeste.br (L.P.O.); henrique.siqueira@ufms.br (H.S.); eloiseambiental@gmail.com (E.S.); 3 Faculty of Engineering, Architecture and Urbanism, University of Western São Paulo, Presidente Prudente 19067175, Brazil; 4 Embrapa Beef Cattle, Brazilian Agricultural Research Corporation, Campo Grande 79106550, Brazil; liana.jank@embrapa.br (L.J.); sanzio.barrios@embrapa.br (S.B.); cbdovalle@gmail.com (C.V.); rosangela.simeao@embrapa.br (R.S.); camilo.carromeu@embrapa.br (C.C.); mateus.santos@embrapa.br (M.S.); 5 Embrapa Instrumentation, Brazilian Agricultural Research Corporation, São Carlos 13560970, Brazil; lucio.jorge@embrapa.br

**Keywords:** deep learning, forage dry matter yield, high-throughput phenotyping, Brazilian pasture

## Abstract

Forage dry matter is the main source of nutrients in the diet of ruminant animals. Thus, this trait is evaluated in most forage breeding programs with the objective of increasing the yield. Novel solutions combining unmanned aerial vehicles (UAVs) and computer vision are crucial to increase the efficiency of forage breeding programs, to support high-throughput phenotyping (HTP), aiming to estimate parameters correlated to important traits. The main goal of this study was to propose a convolutional neural network (CNN) approach using UAV-RGB imagery to estimate dry matter yield traits in a guineagrass breeding program. For this, an experiment composed of 330 plots of full-sib families and checks conducted at Embrapa Beef Cattle, Brazil, was used. The image dataset was composed of images obtained with an RGB sensor embedded in a Phantom 4 PRO. The traits leaf dry matter yield (LDMY) and total dry matter yield (TDMY) were obtained by conventional agronomic methodology and considered as the ground-truth data. Different CNN architectures were analyzed, such as AlexNet, ResNeXt50, DarkNet53, and two networks proposed recently for related tasks named MaCNN and LF-CNN. Pretrained AlexNet and ResNeXt50 architectures were also studied. Ten-fold cross-validation was used for training and testing the model. Estimates of DMY traits by each CNN architecture were considered as new HTP traits to compare with real traits. Pearson correlation coefficient *r* between real and HTP traits ranged from 0.62 to 0.79 for LDMY and from 0.60 to 0.76 for TDMY; root square mean error (RSME) ranged from 286.24 to 366.93 kg·ha${}^{-1}$ for LDMY and from 413.07 to 506.56 kg·ha${}^{-1}$ for TDMY. All the CNNs generated heritable HTP traits, except LF-CNN for LDMY and AlexNet for TDMY. Genetic correlations between real and HTP traits were high but varied according to the CNN architecture. HTP trait from ResNeXt50 pretrained achieved the best results for indirect selection regardless of the dry matter trait. This demonstrates that CNNs with remote sensing data are highly promising for HTP for dry matter yield traits in forage breeding programs.

## 1. Introduction

Pastures are the best alternative for feeding beef cattle in Brazil since they are the most economical and sustainable feed strategy available for cattle rearers. It is estimated that 86% of the beef produced in the country occurs entirely on pastures [1], which are composed mostly of perennial African species such as guineagrass (*Megathyrsus maximus*, syn. *Panicum maximum*) and brachiariagrass (*Urochloa* spp., syn. *Brachiaria* spp.) [2]. Beef cattle production in Brazilian pastures has been increased by the adoption of improved forage cultivars [3]. As an example, the first two guineagrass cultivars released by Embrapa (Brazilian Agricultural Research Corporation) in the 1990s presented 86% (cv. Tanzânia) and 136% (cv. Mombaça) higher leaf dry matter yield than the oldest cv. Colonião with a great impact on the beef and milk production systems [4]. Thus, new improved cultivars are highly recommended to increase profits and sustainability of beef cattle production.

Dry matter yield (DMY) is the most important trait in forage breeding since it contains most of the essential nutrients of the cattle’s diet, such as carbohydrates, proteins, lipids, vitamins, and minerals [5]. Forage dry matter is composed of different morphological components such as leaf blades, leaf sheaths, and stems, and most of the nutrients accumulate in the leaf blades. In all the phases of cultivar development, DMY is evaluated and included in the breeder’s selection criteria. However, the high labor costs and the time spent to acquire DMY phenotypes limit the genetic gains in breeding programs [6].

Currently, phenotyping for forage yield traits is performed by low-cost inaccurate visual evaluations [7] or by high-cost low throughput measurements [8]. For the latter strategy, samples are air-forced dried in a drying chamber to calculate DMY. If breeders seek to include DMY components in the selection criteria, an additional laboring step for separating different components of the forage samples is necessary prior to drying. Additionally, the multi-season x year x environment harvests increase labor costs and time of phenotyping in perennial forage breeding. In this regard, new strategies that guarantee high throughput, accuracy, low costs, and less time consumption would highly impact forage breeding programs, especially the ones with limited resources.

High-throughput phenotyping (HTP) is an emerging strategy to reduce the bottleneck of phenotyping in breeding programs [9]. HTP is performed in fully automated facilities, growth chambers, or in the field; the latter is expected to impact directly plant breeding since the information correlates better with the real environment of crop production [9]. Generally, field HTP uses unmanned aerial vehicles (UAV) platforms combined with different sensors (RGB, multispectral, hyperspectral, LiDAR) and image processing tools. Thus, features (e.g., physiological vegetation indices) are extracted from the images and correlated with ground-truth data to validate the HTP process.

Highly correlated estimates indicate that HTP may be a tool to support breeder or agricultural specialist’s decisions [10,11,12]. Gebremedhin et al. [6] reviewed different aspects of sensor technologies applied to forage DMY and pointed out that machine learning is an important technique that can be widely applied in data analysis for HTP in large population field trials. Remote sensing in conjunction with robust and intelligent data processing methods has been an alternative used for visual inspection of agricultural landscapes in recent years [13].

Recently, machine learning methods such as deep learning have gained prominence, outperforming traditional methods. Spectral indices [14] are commonly used as input for traditional methods. Deep learning-based algorithms, using raw data, search for patterns necessary for classification or regression, with various levels of representation obtained by the composition of non-linear but straightforward models. Each one transforms the representation that is at a raw level into a higher-level representation, slightly more abstract. There are different types of deep neural networks used for image processing, speech recognition, and language interpretation. Convolutional neural networks (CNNs), which are the most used for remote sensing image processing, are deep networks with convolution and pooling layers [15]. They apply mathematical operations of convolution in vectors that represent images, thus extracting information used for training these networks [16].

Deep learning has the advantage of performing end-to-end learning, whereas conventional machine learning often requires a domain-dependent custom feature extraction process [16]. For instance, Lee et al. [17] presented a data pipeline consisting of several steps to use conventional machine learning to evaluate plant growth. The image is preprocessed and submitted to a superpixel algorithm as a feature extraction technique. Using a conventional machine learning algorithm, Random Forest, the model performed plant segmentation. Additionally, they need to compare the images to evaluate plant growth. Our proposal is much simpler and easier to tackle. We use CNNs that incorporate the feature extraction step in the lower levels of the convolutional layers and direct outputs the target information. Other similar approaches in HTP using conventional machine learning can be found in [18,19,20,21]

The applications of CNNs in agriculture are diverse [22,23]. For example, in rice [24], a set of 500 images were used to identify diseases in the field by adopting CNN inspired by AlexNet [25], and LeNet [26]. In the training part of the neural network, the 10-fold cross-validation strategy was used, and the proposed CNN achieved an accuracy of 95.48% in identifying diseases. Research that served as the inspiration for this investigation addressed the use of images and CNNs to estimate biomass in wheat [27], in which a network was proposed inspired by VGGNet [28] and RGB images. They obtained a high correlation coefficient and a low Root Mean Square Error (RMSE) compared with other techniques, such as Random Forest and Support Vector Machines.

In our previous research [29], green biomass was estimated in a tropical forage experiment applying different already known CNNs, such as AlexNet, ResNet, and VGGNet, in a bank of 330 image patches of guineagrass, taken using RGB sensor embedded in UAV. AlexNet outperformed other architectures obtaining a better correlation and intersection between the real data measured by experts and data predicted by these CNNs. However, based on literature, few studies are found in which convolutional neural networks are applied in forages, and we have not found any research that applies these techniques to estimate forage DMY in a HTP context.

Field HTP using remote sensing and CNNs generate new traits throughout the image detection, classification, and retrieval process. These traits can be used in different selection methods in the breeding program, such as indirect selection [30,31], index selection, or in prediction with genomic selection [32]. In the indirect selection, a secondary trait (e.g., HTP trait) can replace the main trait (e.g., DMY) in selection criteria if the secondary trait is highly heritable and correlated to the main trait [33]. However, if the secondary trait is more suitable to evaluate in large plant populations, as expected with HTP traits, a higher selection intensity can be applied and increase the efficiency of indirect selection [34].

To increase the efficiency of forage breeding programs, it is important to evaluate state-of-the-art methods to estimate parameters correlated to important traits. Our work hypothesis is that deep learning regression-based methods are able to estimate DMY traits using only RGB UAV-based imagery with adequate accuracy for a guineagrass breeding program. The main goal is to propose a CNN regression-based approach to estimate DMY traits using only RGB UAV-based imagery. AlexNet, ResNeXt50, DarkNet53, and two networks proposed recently for related tasks named MaCNN [27] and LF-CNN [35], which compose the state-of-the-art, were investigated in the current work. Different from previous work [29] that assessed green biomass, here we considered the dry matter. Besides, the CNN-based DMY was not assessed only in a traditional mode (RMSE, correlation coefficient, etc.) but also in the statistical genetic analysis. Also, the HTP traits from the best CNN were used to evaluate their performance in the indirect selection method.

## 2. Method

**Figure 1:**
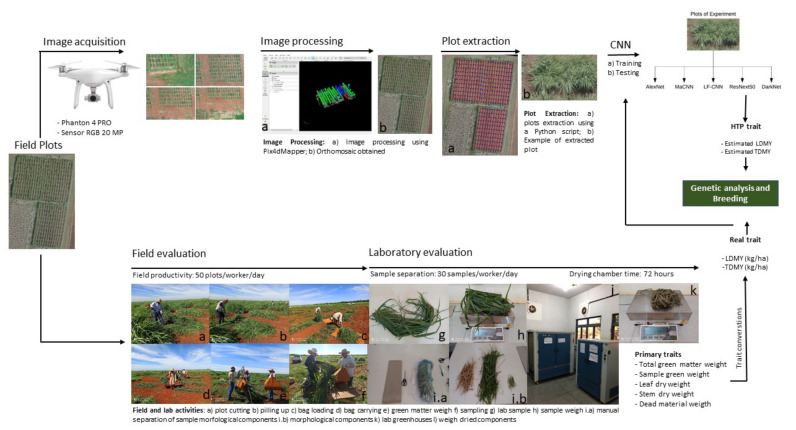
Workflow of phenotyping DMY traits by conventional (**bellow**) and high-throughput (**above**) processes in the guineagrass breeding program at Embrapa Beef Cattle.

The proposed approach is presented in Figure 1. First, RGB images were acquired with a UAV. Orthoimages were generated using the acquired RGB images based on UAV photogrammetry technique. In the next step, the orthoimage for each plot was extracted and used as input for the CNN regression-based architectures. As ground truth, two traits were evaluated for each plot: leaf dry matter yield (LDMY) and total dry matter yield (TDMY), in kg·ha${}^{-1}$. These traits were estimated based on field and laboratory evaluation. Finally, the estimated values from the CNNs were validated on a genetic model. Detailed descriptions of the two main steps are presented in the following sections.

### 2.1. Deep Learning Architectures and Evaluation

AlexNet was the architecture initially chosen for the application. According to the result of previous research focused on the estimation of green matter [29], AlexNet presented the best Pearson correlations and Root Mean Square error, compared to ResNet and VGGNet. AlexNet is a CNN proposed in [25], and is composed of eight layers in which five are convolution layers, using as activation function Rectified Linear Units (ReLU) and MaxPool between the layers, and three layers are fully connected. AlexNet and the other CNNs were adapted to the regression problem.

In addition to this network, two other CNNs containing a number of small layers were chosen for this research. Ma et al. [27] proposed in 2019 a CNN for estimating wheat biomass. Thus, it was decided to use this network and verify its performance to estimate dry matter yield traits in forages. In our study, this network was named MaCNN, and this CNN consists of four convolution layers, with layers between them with the average pooling operation, in which the image dimension is downsampled, thus containing three more levels. In addition, in each convolution layer, a batch normalization was also carried out, which contributes to the acceleration of the CNN training process. Finally, there was a fully connected dropout layer.

Another CNN used was proposed by Barbosa et al. [35] in 2020. This network was named Late Fusion (LF). It is a multi-stream network in which each input is connected to an independent convolutional layer with eight 3 × 3 filters. However, a fully connected ReLU layer with 16 neurons was added to each stream after the maximum cluster layer, followed by a single ReLu neuron. Then, the five neurons (one from each stream) were concatenated and fed to the last two layers.

Even though previous research [29] verified a higher efficiency of AlexNet in relation to deeper networks, it was decided to evaluate state-of-the-art and deeper CNNs for dry matter traits. The architecture ResNeXt [36] with 50 layers was chosen, namely ResNeXt50. In addition to ResNeXt50, the DarkNet53 proposed by Redmon et al. [37] was considered, in which a 53-layer CNN was used. In order to have a better comparison between the different architectures, Table 1 presents the architectures used in this study with the number of layers and the number of parameters.

Data augmentation related to rotation (horizontally and vertically) was also adopted to avoid overfitting, thus increasing the data set for training. For training these architectures, a 10-fold cross-validation technique was used. This procedure randomly divides the data set into 10 sets with equal size, and each set is, in turn, used to test the model trained from the other 10-1 sets [38]. In all experiments, the Adam optimization method [39] was used. The cost function used was the mean square error, described in Equation (1). The pretrained models considered the ImageNet, where the pretrained weights were loaded and adjusted to these models using the training set. Models without pretraining were fully trained using the training set.

(1) $$\varepsilon =\frac{1}{n}\sum_{i=0}^{n}{\left(y_{i}-\hat{y_{i}}\right)}^{2}$$

To evaluate the experiments, different metrics were used, as expressed in Equations (2)–(4). The MAE and RMSE metrics express the average model prediction error in units of the variable of interest. The metric *r* establishes a linear relationship between the real value and the predicted value. In the Equations (2) and (3), the *y* is the real value and the $\hat{y}$ the predicted one. In the last equation, *x* represents real values, and $\bar{x}$ is the average of real values. The *y* represents the predicted values and the $\bar{y}$ the average of the predicted values.

(2) $$MAE=\frac{1}{n}\sum_{i=0}^{n}\left|y_{i}-\hat{y_{i}}\right|$$

(3) $$RMSE=\sqrt{\frac{1}{n}\sum_{i=0}^{n}{\left(y_{i}-\hat{y_{i}}\right)}^{2}}$$

(4) $$r=\frac{\sum_{i=1}^{n}\left(x_{i}-\bar{x}\right)\left(y_{i}-\bar{y}\right)}{\sqrt{\sum_{i=1}^{n}{\left(x_{i}-\bar{x}\right)}^{2}{\left(y_{i}-\bar{y}\right)}^{2}}}$$

Finally, to evaluate the distribution of real and estimated values, we constructed histograms. For the construction of the histograms, the number of bins was determined using the [40] elbow rule from the partitioning of the real values of the samples.

### 2.2. Validation of HTP Traits on a Genetic Model

Each CNN architecture generated estimates of each DMY trait from the cross-validation process for each trial plot. Here, these estimates for a given CNN were nominated HTP traits, which means a trait estimated from a UAV platform-RGB sensor-CNN architecture related to the real trait (LDMY or TDMY). Thus, DMY traits estimated by HTP and by conventional phenotyping were analyzed considering the linear mixed model shown in Equation (5).

(5) $$y=XB+Z_{1}b+Z_{2}g+e$$

where *y* is the vector of observations of the real or each HTP trait; *B* is the vector of fixed effects of replications and checks (genitors and cultivars); *b* is the vector of random effects of blocks within replications where *b ^~^ N(0,Vb)*; *g* is the vector of random effects of full-sib family where *g ^~^ N(0,Vg)*; and *e* is the vector of random effects of residuals where *e ^~^ N(0,RVe)*. *R* is a matrix of (co)variances of residuals where the spacial tendencies were modeled for autocorrelation among lines and columns of the trial according to [41]. X relates *y* to *B*, whereas *Z*_1_ and *Z*_2_ relate *y* to *b* and *g*, respectively. Estimation of variance components from the data was carried out using restricted maximum likelihood (REML) by the Average Information algorithm as implemented in the ASREML-R package [42] in the R environment. Based on the most likely variance components, the fixed effects were estimated, and the random effects (family BLUPs) were predicted by solving the mixed model equations.

To compute the broad-sense heritability (H) for real and HTP traits on the basis of full-sib family means, we considered Equation (6), as suggested by [43]:

(6) $$H=1-\frac{PEV}{2Vg}$$

where *PEV* is the prediction error variance, which represents the average variance of the difference between a pair of family predictions (BLUP), and *Vg* is the genetic variance component among full-sib families.

Correlations between family BLUPs of a given HTP trait and its related real trait (LDMY or TDMY) were estimated as an approximated genetic correlation (*r*) between traits.

Direct response to selection (DR) for real traits and correlated response to selection (CR) when using its related HTP trait as a secondary trait were obtained using Equations (7) and (8) [33], where *i* is the selection intensity, *h* is the square root of H, *v* is the square root of Vg.

(7) $$DR=i.h_{\mathrm{real}}.v_{g\mathrm{real}}$$

(8) $$CR=i.h_{\mathrm{HTP}}.r.v_{g\mathrm{real}}$$

For DR, we considered a selection intensity of 10%, or approximately nine selected families, which represented an *i* = 1.76; for CR we considered three scenarios that maintained the same number of selected families: (1) the same selection intensity (10%) as for the real trait, (2) increasing the selection intensity to 5% or *i* = 2.06 by simulating twice the number of full-sib families evaluated (172) by HTP and (3) increasing the selected intensity to 1% or *i* = 2.67 by simulating ten times the number of full-sib families evaluated (860) by HTP. In the last two scenarios we must consider the same experimental design as for the first one. Thus, the total number of plots considered in (2) and (3) were 660 and 3300, respectively.

## 3. Experiments and Results

### 3.1. Study Area and Data Set

**Figure 2:**
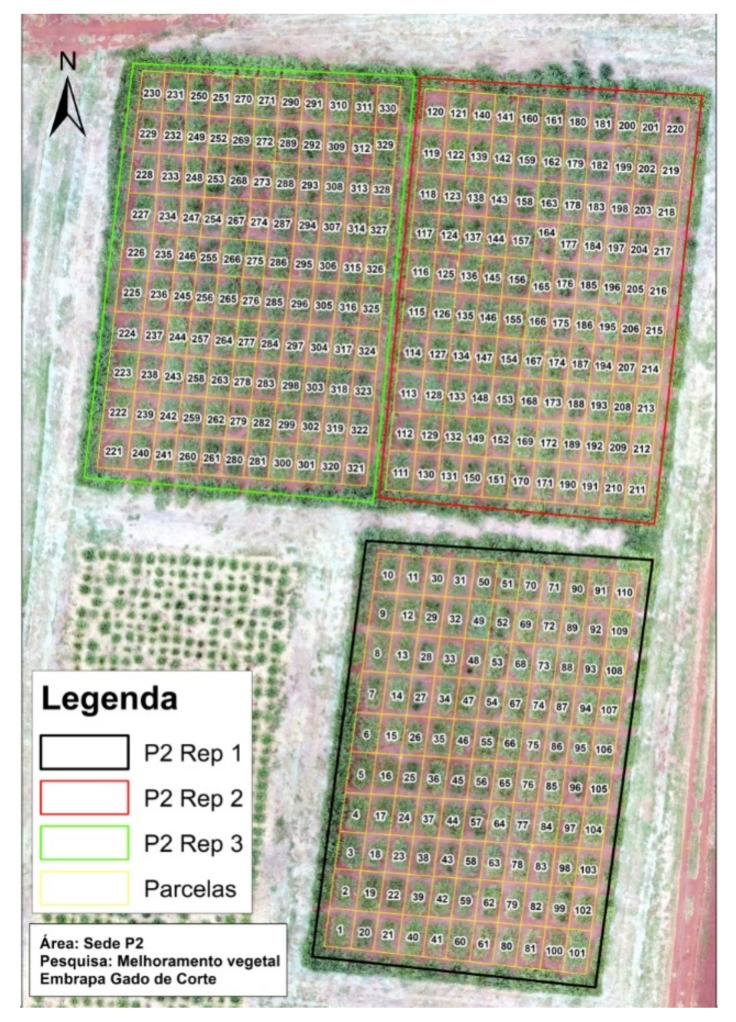
Sketch of the trial in the field.

For the development of this study, images from a guineagrass trial located at Embrapa Beef Cattle in Campo Grande, Mato Grosso do Sul, Brazil, were used. A total of 110 genotypes composed of 86 full-sib families, ten sexual, and ten apomictic genitors along with four commercial checks (Mombaça, MG12 Paredão, BRS Quênia, and BRS Tamani) were used as treatments. A 10 × 11 alpha lattice design with three replications was considered, totaling 330 plots in the trial. Each plot consisted of two rows of 2.0 m length and 0.5 m apart. Each row consisted of five plants spaced 0.5 m between plants, in a total of ten plants per plot. Plots were 1.0 m apart, representing an area of 4.5 m${}^{2}$. This trial is described according to Figure 2.

The images were taken using the UAV Phantom 4 PRO, with an RGB sensor of 5427 × 3748 of image resolution. The flight was carried out on 23 January 2019, close to 9 am, at the height of 18 m, thus generating a resolution of 0.5 cm/pixel, with a frontal image overlap of 75% and lateral of 60%, and the flight took about 20 min.

To properly extract each plot, the images were processed using the software Pix4dMapper. An orthomosaic was generated, which is a mosaic of orthorectified and enhanced aerial images to homogenize its appearance. With the orthomosaic generated, it was possible to correctly map the area of interest and perform the extraction of images from each plot of the trial. To perform this extraction, a Python script proposed in [29] was used, in which it uses the orthomosaic as a parameter in tiff format and from the information of the trial, such as the number of blocks and the number of plots per block, as well as how the numbering of each plot is organized, generates the images of each plot identified according to the established numbers. For this experiment, 330 orthoimage patches were generated relative to the total number of plots in the trial.

Each plot was evaluated for forage yield traits on 25 January 2019. Traits were obtained by harvesting each plot 0.2 m from the soil. The harvested material (green matter) was weighed in the field using a field dynamometer to obtain the total green matter weight per plot (TGMW) in kg. After weighing, samples of 300 g to 500 g of the green matter were taken and sent to the Laboratory of Forage Sample Preparation of Embrapa Beef Cattle to obtain the sample green weight (SGW), in g, and which were then separated into leaf blades, leaf sheaths + stems and dead material. These forage sample components were then air-forced dried at 65 ${}^{{}^{\circ}}$C in the drying chamber for 72 h. After this period, the dried samples were weighed to obtain leaf dry matter weight (LDMW), sheaths + stem dry matter weight (SSDMW), and dead material dry matter weight (DMDMW) in kg. The Equations (9)–(12) were considered to obtain the dry matter yield per plot.

(9) $$LDMY=\frac{TGMW*LDMW}{SGW}$$

(10) $$SSDMY=\frac{TGMW*SSDMW}{SGW}$$

(11) $$DMDMY=\frac{TGMW*DMDMW}{SGW}$$

(12) TDMY=LDMY+SSDMY+DMDMY

Finally, LDMY and TDMY per plot were converted to kg·ha${}^{-1}$.

**Figure 3:**
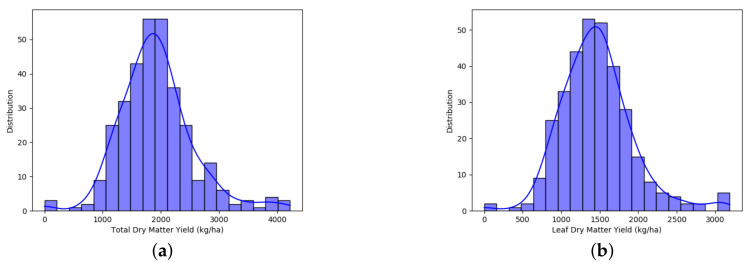
Data distribution. (**a**) TDMY; (**b**) LDMY.

The distribution of the values of LDMY and TDMY in kg·ha${}^{-1}$, which for this research will be the attributes classes *y*, are shown in Figure 3.

### 3.2. Deep Learning Protocol

Due to the fact that only 330 image patches were used, this can become a problem when using deep neural networks, as there is a high number of parameters that are estimated. As previously mentioned, data augmentation related to rotation (horizontally and vertically) was also adopted to avoid overfitting, thus increasing the data set for training.

For training the architectures, a 10-fold cross-validation technique was used. The Adam optimization method [39] was used, with the descending gradient algorithm, and a fixed learning rate of 0.001, constant $\beta_{1}=0.9$, $\beta_{2}=0.999$ and $\epsilon =10^{-8}$. Table 2 summarizes the CNN architectures used, the number of epochs for each experiment, and the batch size. The number of epochs for each experiment was defined empirically using early stopping evaluated every 100 epochs. The batch size sought to use the highest value that did not overflow the GPU memory. For all experiments, a server with an NVIDIA K80 (2 × 12 GB), AMD TR 1900X 3.7 GHz CPU, and 64 GB of RAM was used.

### 3.3. Results

Table 3 and Table 4 present the MAE, the RMSE and the Pearson Correlation coefficient *r* for each CNN architecture with respect to the dry matter traits. From the tables presented, the MAE values ranged between 204.39 (AlexNet pretrained) and 266.77 (LF-CNN) kg·ha${}^{-1}$ for the LDMY trait, and ranged between 289.66 (AlexNet pretrained) and 366.93 (LF-CNN) kg·ha${}^{-1}$ for TDMY trait. It is important to mention that we have values, predominantly, from 500 to 4000 kg·ha${}^{-1}$ for these traits. Therefore, it is possible to analyze a better performance of AlexNet pretrained for LDMY trait, since it presented the lowest values for MAE and RMSE, with an absolute average error of 204.39 kg·ha${}^{-1}$. For TDMY trait, AlexNet pretrained presented the lowest MAE in relation to the others; however, for the other metrics, ResNeXt50 pretrained presented better results, with an RMSE of 413.07 kg·ha${}^{-1}$.

Furthermore, analyzing the coefficient *r*, we verified that there was a variation from 0.62 (LF-CNN) to 0.79 (AlexNet pretrained) for LDMY and 0.60 (LF-CNN) up to 0.76 (ResNeXt50 pretrained) for TDMY. This shows that there was a significant correlation between the data estimated by the CNN and the real data obtained in the field, realizing that it is possible to obtain a high relationship between the RGB images and the dry matter data using the CNN. Overall, the three networks with the best results were AlexNet pretrained, ResNeXt50 pretrained, and DarkNet53. LF-CNN had the worst results for both traits.

#### 3.3.1. Graph of Predicted Versus Real

**Figure 4:**
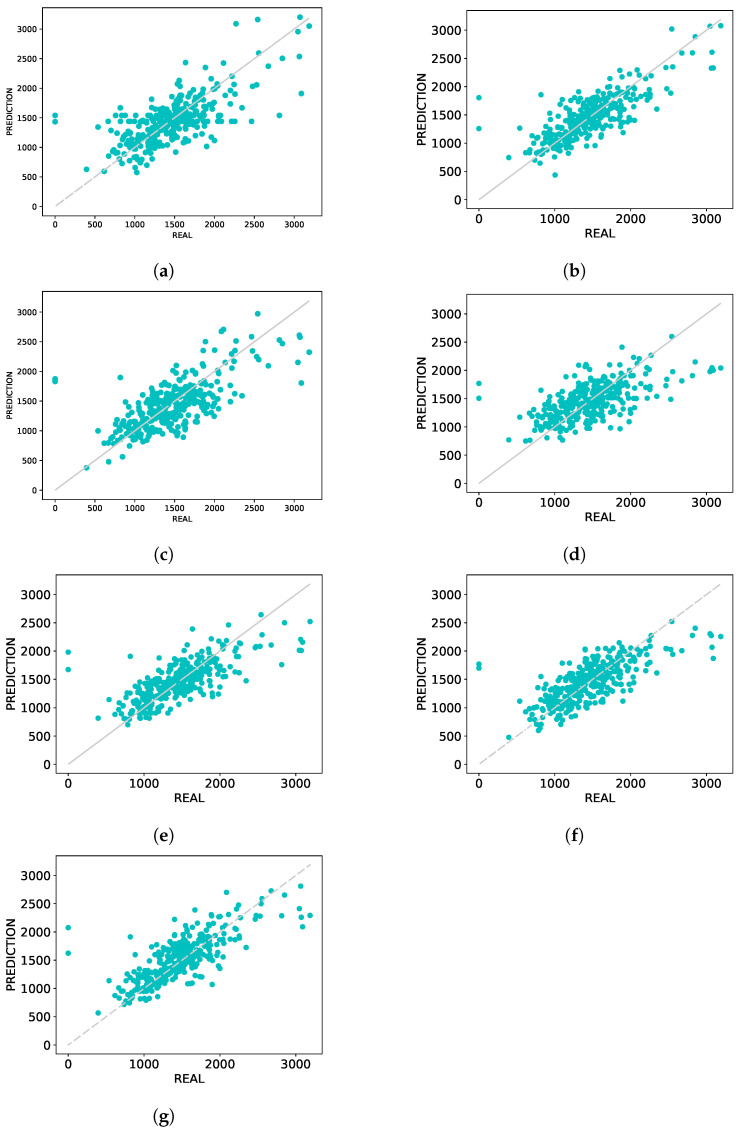
Predicted vs. Real plots-LDMY. (**a**) AlexNet; (**b**) AlexNet pretrained; (**c**) MaCNN; (**d**) LF-CNN; (**e**) ResNeXt50; (**f**) ResNeXt50 pretrained; (**g**) DarkNet53.

**Figure 5:**
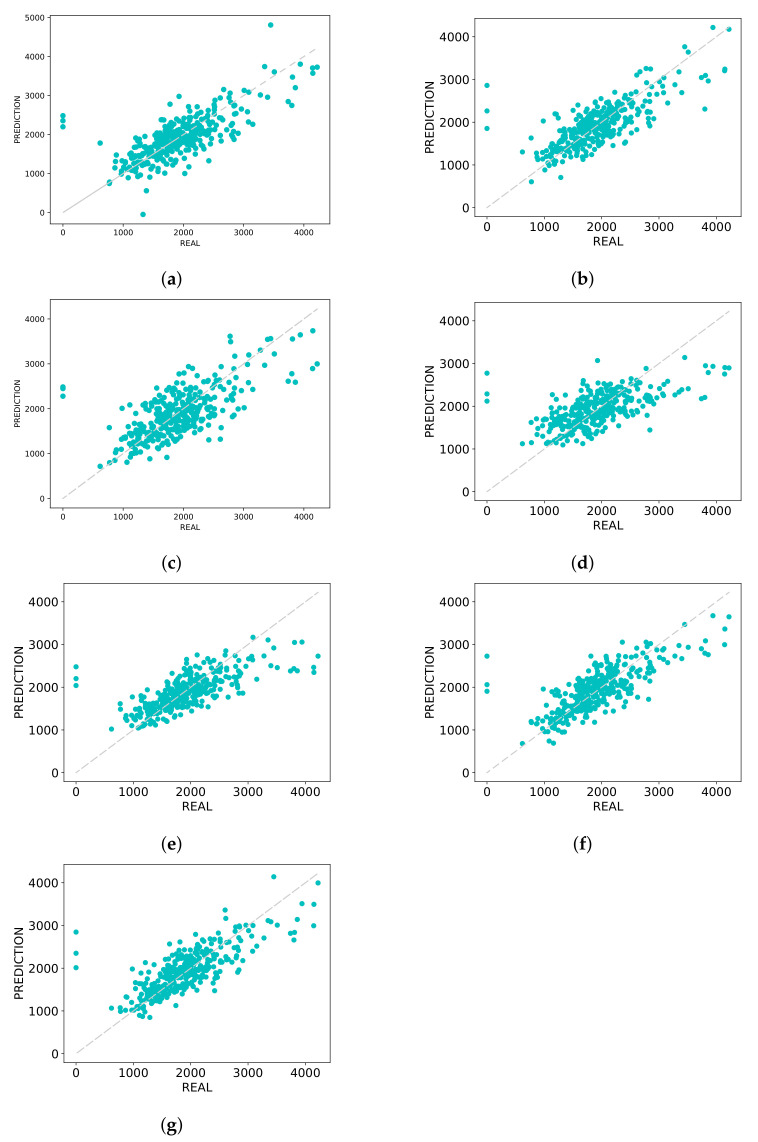
Predicted vs. Real plots-TDMY. (**a**) AlexNet; (**b**) AlexNet pretrained; (**c**) MaCNN; (**d**) LF-CNN; (**e**) ResNeXt50; (**f**) ResNeXt50 pretrained; (**g**) DarkNet53.

The graphs shown in Figure 4 and Figure 5 are composed of points $\left(y,\hat{y}\right)$, in which *y* represents the real values of LDMY and TDMY, respectively, while $\hat{y}$ represents the values predicted by CNNs for each of the traits. A perfect prediction would result in a straight line on the graph since the predicted, and real values would be the same.

Analyzing the graphs presented in Figure 4, which presents the plot of the estimated and real values of LDMY, it is possible to notice that for high values, above 2500 kg·ha${}^{-1}$, AlexNet pretrained (Figure 4b) provided smaller errors. The ResNeXt50 pretrained and DarkNet53 models presented more linear graphs (Figure 4f,g), providing less error mainly in the range of 1000 and 2000 kg·ha${}^{-1}$. The other networks (Figure 4a,c–e) have some major errors with respect to values between 1000 and 2000 kg·ha${}^{-1}$, and for higher values, they were able to estimate the values properly.

For TDMY, the analysis of the graphs presented in Figure 5 occured in a similar way. For this trait, we can notice that AlexNet pretrained (Figure 5b), ResNeXt50 pretrained (Figure 5f) and DarkNet53 (Figure 5g) provided more accurate results, mainly for values above 3000 kg·ha${}^{-1}$. The MaCNN (Figure 5c) and LF-CNN (Figure 5d) networks presented small errors for values between 1000 and 2000 kg·ha${}^{-1}$, but they started to disperse more as the values increased (between 2000 and 3000 kg·ha${}^{-1}$). However, MaCNN in some data had a smaller error for values above 3000 kg·ha${}^{-1}$, which was not verified for LDMY. Finally, we verified that AlexNet not pretrained had more spread points for values below 1500 kg·ha${}^{-1}$. On the other hand, ResNeXt50 not pretrained presented more spread points for values above 3000 kg·ha${}^{-1}$.

#### 3.3.2. Histograms

**Figure 6:**
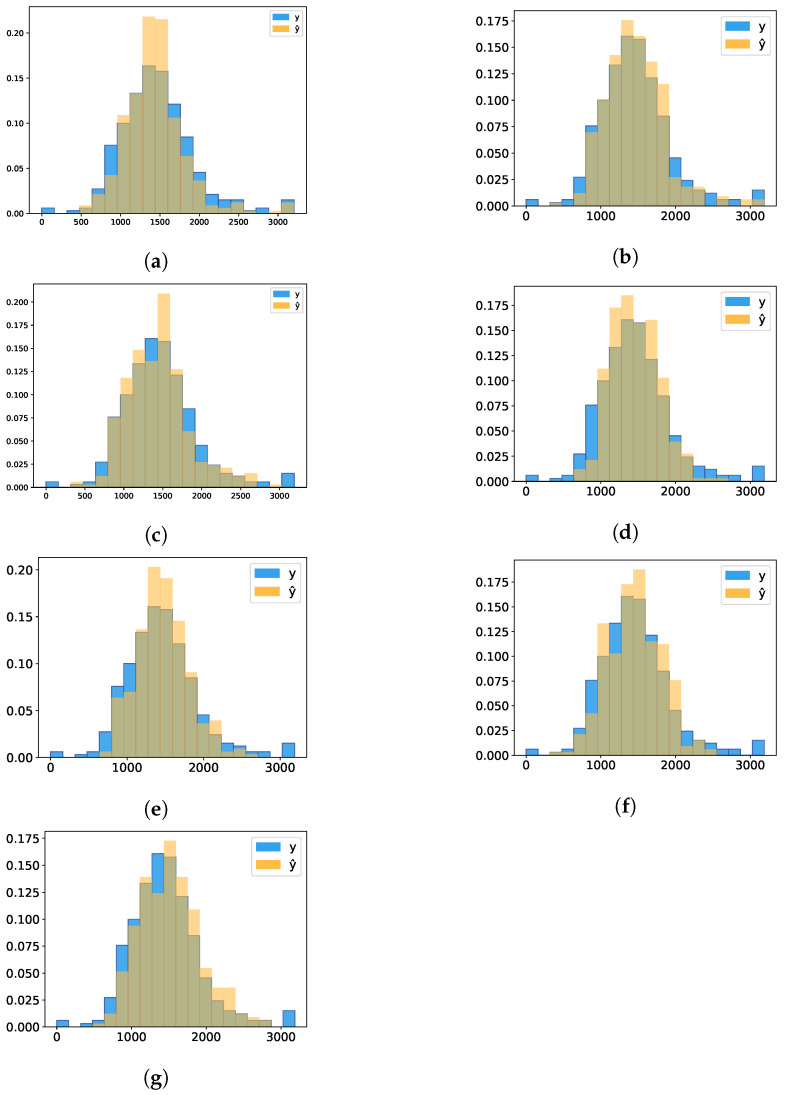
Comparison of predicted $\hat{y}$ vs. real *y* in relation to LDMY data distribution. (**a**) AlexNet; (**b**) AlexNet pretrained; (**c**) MaCNN; (**d**) LF-CNN; (**e**) ResNeXt50; (**f**) ResNeXt50 pretrained; (**g**) DarkNet53.

**Figure 7:**
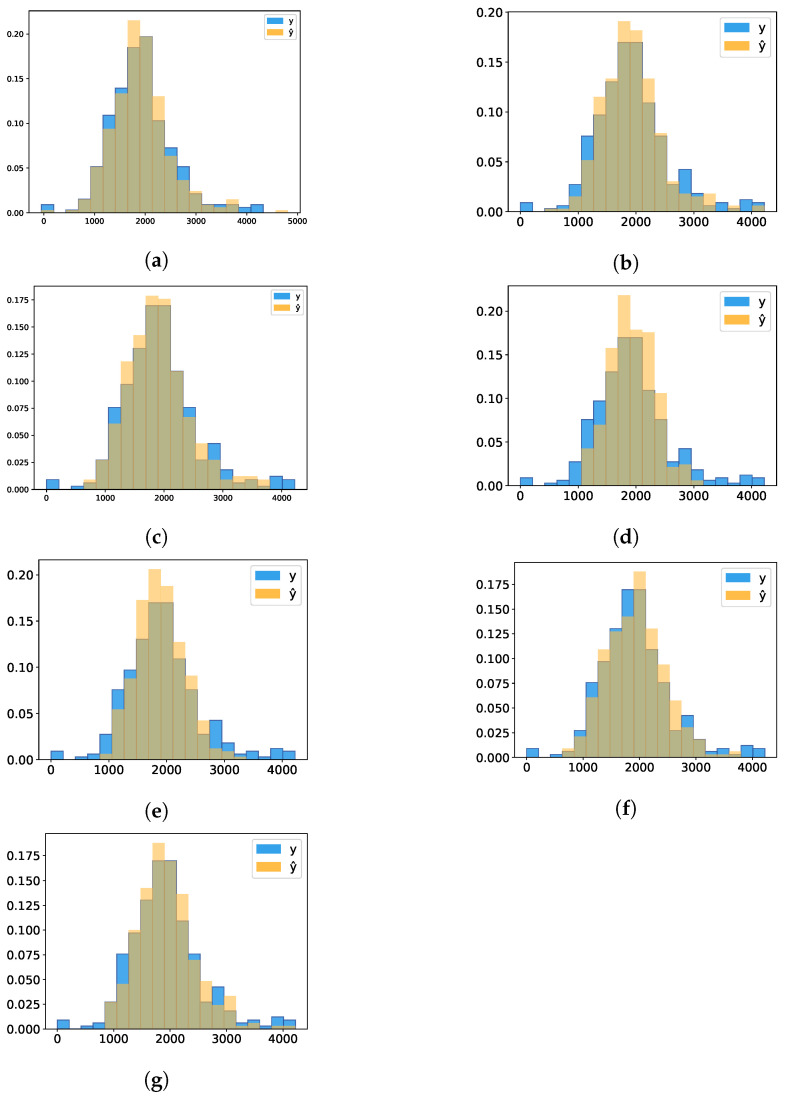
Comparison of predicted $\hat{y}$ vs. real *y* in relation to TDMY data distribution. (**a**) AlexNet; (**b**) AlexNet pretrained; (**c**) MaCNN; (**d**) LF-CNN; (**e**) ResNeXt50; (**f**) ResNeXt50 pretrained; (**g**) DarkNet53.

The histograms (Figure 6 and Figure 7) show the distribution of data of LDMY and TDMY, respectively, and the intersection between the predicted values $\hat{y}$ and real values *y*. The intersection area between the values was then calculated and is presented in Table 5. It was verified that for group values greater than 20, the addition of new groups did not significantly increase the representativeness of the data; thus, the number of bins was defined as 20.

From the analysis of the tables and the histograms presented above, it is possible to notice a higher area of intersection for AlexNet.

#### 3.3.3. Genetic Parameters and Indirect Selection Efficiency

Results in Table 6 showed that the V${}_{g}$ components changed according to the HTP trait considered, but all of them were lower than for the Real LDMY and TDMY traits. It can be observed that traits derived from ResNeXt50 pretrained showed 5.5 and 3.8 times higher *Vg* than those derived from LF-CNN for LDMY and AlexNet for TDMY, respectively. Compared to the Real traits, the V${}_{g}$ of ResNeXt50 pretrained were 0.66 and 0.73 times of the V${}_{g}$ for LDMY and TDMY, respectively. Interestingly, ResNeXt50 pretrained increased the estimates of *Vg* in comparison to ResNeXt50, a non-pretrained model, especially for TDMY. DarkNet53 was another CNN that stood out for estimating the *Vg* of the traits compared to the other networks.

Broad-sense heritability (*H*), which is related to the accuracy of phenotypes to predict genotypes, ranged from −0.49 (LF-CNN) to 0.45 (ResNeXt50 pretrained) for LDMY and from −0.36 (AlexNet) to 0.51 (ResNeXt50 pretrained) for TDMY, indicating that there were effects of the phenotyping process in the heritability of the traits. Except for HTP traits from ResNeXt50 (only for TDMY), ResNeXt50 pretrained, and DarkNet53, all the other CNN generated traits with lower *H* than real traits. Thus, while some CNN generated phenotypes with moderate accuracy to predict the family genotypic values, other CNN showed low or even highly inaccurate values, as in the case of LF-CNN for LDMY and AlexNet for TDMY.

Genotypic correlations (*r*) between HTP and real traits were positive and high, although their magnitudes varied according to the CNN architecture. The values ranged from 0.71 (AlexNet and LF-CNN) to 0.85 (AlexNet pretrained) for LDMY and from 0.69 (MaCNN) to 0.85 (DarkNet53) for TDMY. The highest correlations were achieved by HTP traits generated by AlexNet pretrained, DarkNet53 and ResNeXt50 pretrained for LDMY and DarkNet53, ResNext50 pretrained, and AlexNet for TDMY.

ResNeXt50 pretrained was chosen to estimate the efficiency of indirect selection using HTP secondary traits, since it showed a generally better performance for *Vg*, *H* and *r* for both dry matter traits. Table 7 shows that the CR/DR ratios were 0.88 and 0.86 for LDMY and TDMY, respectively, when considering the same number of plots (330), population size (86) and selection intensity (10%). This means that the CR was 88% and 86% of the efficiency of DR for LDMY and TDMY, respectively.

Simulated scenarios shown in Table 7 compare the selection response using the HTP process in larger trial and population sizes with conventional process maintaining the current trial and population size. It is important to mention that the estimated selection efficiency would only be achieved if the same experimental design were to be repeated, since the genetic parameters are considered the same regardless of the population size. If selection intensity increased to 5% by evaluating 660 plots (172 full-sib families) in the field, the CR/DR ratio would be 1.03 for LDMY and 1.00 for TDMY, indicating that CR would be 103% of the DR for LDMY and similar for TDMY. The CR might be efficient if we applied a selection intensity of 1% by evaluating 3300 plots (860 full-sib families) instead. In this latter scenario, the CR/DR ratio would be 1.34 for LDMY and 1.30 for TDMY, which means that the CR would be 134 and 130% of the DR for these DMY traits.

## 4. Discussion

The results showed that the CNNs with the best performance for the estimate of the LDMY and TDMY were the AlexNet pretrained, ResNeXt50 pretrained, and DarkNet53. Regarding the statistical-genetic analysis, it was noticed that ResNeXt50 pretrained and DarkNet53 were the best CNNs to estimate genetic parameters. The literature usually reports deeper networks with better performance than shallower networks [44,45]. For our final aim, which is related to the genetic analysis, we also verified that deeper networks provided more accurate solutions.

The results for the LDMY and TDMY traits showed a high correlation with the ground truth, in general, higher than 0.75 for the three best CNNs. This indicates that the high-throughput and the conventional phenotyping processes were highly correlated in this study. The result presented in Ma et al. [27] to estimate the above-ground biomass in winter wheat showed an R${}^{2}$ correlation of 0.80, which is slightly greater than that obtained in our investigation. To compare the results with their study, we used the same network to estimate dry matter in guineagrass, but it did not achieve the same performance presented in the paper. In the research of [46], using RGB images and crop surface models to estimate dry matter in barley, they obtained an RMSE between 97 and 234 g·m${}^{-2}$, while this experiment had an RMSE between 41.3 to 50.65 g·m${}^{-2}$.

Regarding the correlation between predicted and real data, it was possible to verify that, to estimate dry matter yield traits, the RGB images obtained with UAV and the CNNs can have a high correlation. However, as presented in the previous research [29], the correlation was better for estimating green than dry biomass. A possible explanation is that the image presented to the neural network, which represents the plot before harvest, is more related to the green matter yield. Thus, the dry matter obtained in the laboratory process was not fully represented in the RGB images, thus making extracting characteristics through the network more laborious. Nevertheless, the performance obtained was encouraging, even when compared with other methodologies for estimating biomass.

From the statistical-genetic analysis, it was noticed that ResNeXt50 pretrained and DarkNet53 were the best CNNs to estimate genetic variance and heritability. Estimates from AlexNet pretrained failed to exploit these parameters, especially the heritability, thus showing the importance of the validation of the CNN in a genetic model. Our results showed that the HTP traits generated by these networks presented a larger *Vg* and higher *H* when compared to the other CNN. When compared to the Real traits, it showed lower *Vg* and a similar *H*. Thus, while the accuracy of the high-throughput process was comparable to the conventional process, it failed to exploit the available genotypic variance among families. This situation is expected due to the small number of examples to train the model, mainly for data in the extreme of the normal distribution, as shown in Figure 3. Thus, to overcome this limitation, we expect that increasing the size as well as the variability of examples of the data set will increase the power of ResNeXt50 and DarkNet53 to estimate phenotypes more representative of the genetic variability.

Theoretically, selection for one trait will cause a correlated response to selection in a second trait if a genetic correlation exists between the two traits [33]. This is an important concept in applying HTP traits in indirect selecion in plant breeding. Our results indicated high genetic correlation (*r* > 0.69) between the HTP and Real traits even for CNN with lower performance for the other genetic parameters. This indicates the high potential of HTP process (UAV-RGB-CNN) used in this research to be considered in different strategies in guineagrass breeding for DMY traits. The CNN, AlexNet pretrained, ResNeXt50 pretrained and DarkNet53 stood out among the other CNN showing the highest values of *r* (>0.81). Thus, the results of genotypic correlations are in accordance with the results shown by the standard evaluations of the algorithms. From a selection point of view, high correlations indicate that the ranking of the family genotypic values were highly coincident between HTP and Real traits, and the best-selected families would also be highly coincident.

Indirect selection is one of the main applications of HTP in breeding programs [32]. The potential of HTP in improving the efficiency of early generation selection has been studied in sugarcane [30], and wheat [31]. In both studies, the results were encouraging to use HTP traits and indirect selection. In our results with guineagrass, considering the same selection intensity for DR and CR (*i* = 10%), the efficiency of indirect selection (CR/DR ratio) was 88 and 86% of the direct selection for LDMY and TDMY traits, respectively. These lower indirect selection efficiencies for the DMY traits were due to the lower magnitudes of the product between the heritability of the HTP traits (*h*_HTP_) and the genetic correlation between the HTP and the real traits (*r*) compared to the heritability of the real trait (*h*_real_). Natarajan et al. [30], using indirect selection based on NDVI as HTP trait, reached 73% of the efficiency of direct selection for yield in sugarcane. Also, using NDVI, Krause et al. [31] reported a higher efficiency of indirect selection when compared to visual selection for grain yield in lines of wheat in early generations. Thus, these results in different crops show that HTP is a promising strategy to improve the efficiency of breeding programs.

Increasing the size of the breeding program to enable higher selection intensity is one of the main benefits of using HTP [34]. This important aspect of HTP stimulated us to simulate scenarios where the only parameter to be changed is the selection intensity. This is achieved by increasing the number of plots and families evaluated and maintain the heritability and genotypic variance of HTP traits unchanged. Our results showed a high increase in the indirect selection efficiencies for DMY traits were achieved when multiplying by two (100 to 103%) and by ten (130 to 134%) times the size of the population evaluated in the field by the high throughput process using ResNeXt50 pretrained. Here the CR/DR ratio increased due to the higher values of *i* (2.06 for *i* = 5% and 2.67 for *i* = 1%) applied in larger populations evaluated by the proposed process. This means that families with superior DMY values are more likely to be selected in a larger population evaluated with the HTP process. Although these results are promising, they are not yet useful to extend to different populations or environments (regions/climates). In further researches, we will include data from a wider range of environments (seasons, locations) to investigate the generalization of the methodology to a diversity of scenarios. Investigation regarding domain adaptation will also be performed.

Resources aspects as labor, time and cost are important phenotyping issues for breeding programs, since evaluations are performed in a range of environments, seasons and populations. Thus, we compared these issues between conventional and high-throughput phenotyping processes (Figure 1) used in guineagrass breeding program. We used LDMY trait for comparison since it requires all steps of phenotyping in the field and in the laboratory. Conventional phenotyping took about eight days to obtain phenotypes: one day for field evaluations (seven workers), three and a half days for sample separation in the laboratory (three workers), three days for samples drying in the dry chamber, and a half-day for dried samples weighing and preparing the data for genetic analysis (one worker). With the HTP process, it took us about four and a half hours as following: 13 min to UAV flight covering an area of 1.5 ha, four hours for the process to generate the orthomosaic, approximately 10 min, based on the period of organizing the data to use in the algorithm and the execution of it for extracting plots, which is very fast, approximately 15 s to apply the ResNeXt50 pretrained algorithm, considering it was just trained. Therefore, we see here that the HTP process with ResNeXt50 pretrained can produce phenotypes with the same accuracy with less time (a half-day) labor and cost, when compared to the conventional process.

If the size of the program were increased by ten times, as suggested here to increase until 34% the response to selection, the conventional phenotyping process for DMY traits would be impracticable for the current resources available for the guineagrass breeding program. But it would be viable using the HTP phenotyping process, due to the advantages just presented. Resources saved by using the high-throughput phenotyping would be reallocated to the management of activities related to the increased breeding program size and the infrastructure necessary for HTP, as UAV, sensor, and computation services.

HTP based on UAV brings some technical challenges related to working conditions (e.g., sun angle, wind, clouds) that alter the image quality. Also, there are some UAV-related problems such as flight safety, flight time, and limited payload [15,47]. Additionally, the image overlap associated with the pasture features on images can cause errors during the orthomosaic generation. Forage breeding images obtained from UAV are very similar, making the matching process difficult. An overlap higher than 80% (front and side) is recommended to reduce gaps in the generated orthoimages.

## 5. Conclusions

The high-throughput phenotyping has been increasingly used in research to improve different species, as well as tropical forages. Thus, it is possible to conclude that remote sensing with low cost unmanned aerial vehicles embedded with high-resolution RGB sensors, together with convolutional neural networks, is a promising technique to be used to estimate dry matter yield in the guineagrass breeding program. Moreover, the ResNeXt50 with pretraining shows the best results since this network is able to estimate more accurately the genetic parameters. In future investigations, we expect to increase the data set and its variability by evaluating other experimental fields with other environmental characteristics. Finally, when performing these procedures, it is expected that CNN’s robustness will be optimized and, with this, it will be applied as a tool for increase the efficiency of selection in forage breeding programs.

## Figures and Tables

**Table 1:** Comparison between the different models in terms of number of layers and parameters.

Models	Number of Layers	Number of Parameters
AlexNet	8	62 M
AlexNet pretrained	8	62 M
MaCNN	5	1.1 M
LF-CNN	10	3.6 K
ResNeXt50	50	25 M
ResNeXt50 pretrained	50	25 M
DarkNet53	53	42 M

**Table 2:** Configurations of the experiments.

Models	Number of Epochs	Batch Size
AlexNet	500	256
AlexNet pretrained	500	256
MaCNN	500	256
LF-CNN	500	256
ResNeXt50	300	64
ResNeXt50 pretrained	300	64
DarkNet53	300	64

**Table 3:** Results for LDMY.

Models	Mean Absolute Error	Root Mean Square Error	Pearson Correlation (*r*)
AlexNet	248.41 ± 47.58	340.70 ± 64.85	0.70 ± 0.09
AlexNet pretrained	**204.39 ± 56.46**	**286.24 ± 80.39**	**0.79 ± 0.12**
MaCNN	240.74 ± 65.09	333.60 ± 86.93	0.71 ± 0.11
LF-CNN	266.57 ± 89.19	366.93 ± 110.33	0.62 ± 0.13
ResNeXt50	221.04 ± 54.44	319.98 ± 98.47	0.72 ± 0.12
ResNeXt50 pretrained	231.66 ± 63.41	319.58 ± 87.06	0.73 ± 0.10
DarkNet53	217.30 ± 57.09	311.76 ± 76.68	0.76 ± 0.12

**Table 4:** Results for TDMY.

Models	Mean Absolute Error	Root Mean Squared Error	Pearson Correlation (*r*)
AlexNet	311.37 ± 88.58	441.31 ± 131.29	0.73 ± 0.17
AlexNet pretrained	**289.66 ± 96.28**	419.95 ± 136.93	0.75 ± 0.20
MaCNN	345.11 ± 97.94	477.12 ± 136.34	0.68 ± 0.20
LF-CNN	364.44 ± 145.38	506.56 ± 176.24	0.60 ± 0.17
ResNeXt50	306.09 ± 137.15	449.07 ± 175.06	0.71 ± 0.17
ResNeXt50 pretrained	294.73 ± 78.83	**413.07 ± 117.77**	**0.76 ± 0.24**
DarkNet53	291.12 ± 80.26	419.50 ± 131.87	0.75 ± 0.23

**Table 5:** Intersection area of histograms.

Experiments/Intersection	Leaf Dry Matter	Total Dry Matter
AlexNet	0.87	**0.93**
AlexNet pretrained	**0.91**	0.90
MaCNN	0.89	0.91
LF-CNN	0.86	0.81
ResNeXt50	0.88	0.85
ResNeXt50 pretrained	0.86	0.89
DarkNet53	0.89	0.90

**Table 6:** Genetic parameters for dry matter traits in the guineagrass breeding program using field data and their estimates by several CNNs architectures.*V${}_{g}$*-Genetic variance among full-sib families; H-broad sense heritability; r-approximate genetic correlation between real and HTP trait.

Data Origin	Traits					
	Leaf Dry Matter Yield			Total Dry Matter Yield		
	*V* ${}_{g}$	*H*	*r*	*V* ${}_{g}$	*H*	*r*
Real	44,258	0.41	1.00	87,562	0.48	1.00
AlexNet	18,299	0.08	0.71	16,956	−0.36	0.82
AlexNet pretrained	15,602	0.05	0.85	51,343	0.11	0.81
MaCNN	18,643	0.08	0.76	32,888	0.10	0.69
LF−CNN	5288	−0.49	0.71	23,975	0.23	0.75
ResNeXt50	21,188	0.36	0.78	37,580	0.47	0.80
ResNeXt50 pretrained	29,337	0.45	0.84	64,288	0.51	0.83
DarkNet53	22,347	0.44	0.84	62,378	0.45	0.85

**Table 7:** Direct (DR) and correlated (CR) response to selection for dry matter traits in the guineagrass breeding program using field and data estimated by the ResNeXt50 pretrained CNN architecture. SI—selection intensity.

Responses to Selection	Trait	
	Leaf Dry Matter Yield (kg·ha${}^{-1}$)	Total Dry Matter Yield (kg·ha${}^{-1}$)
	330 plots	
DR (SI = 10%)	237	361
CR (SI = 10%)	209	309
CR/DR	0.88	0.86
	660 plots	
CR (SI = 5%)	244	361
CR/DR	1.03	1.00
	3300 plots	
CR (SI = 1%)	317	468
CR/DR	1.34	1.30

## Data Availability

Not applicable.
